# Comprehensive Analysis of Common Serum Liver Enzymes as Prospective Predictors of Hepatocellular Carcinoma in HBV Patients

**DOI:** 10.1371/journal.pone.0047687

**Published:** 2012-10-24

**Authors:** Hie-Won Hann, Shaogui Wan, Ronald E. Myers, Richard S. Hann, Jinliang Xing, Bicui Chen, Hushan Yang

**Affiliations:** 1 Division of Gastroenterology and Hepatology, Department of Medicine, Liver Disease Prevention Center, Thomas Jefferson University, Philadelphia, Pennsylvania, United States of America; 2 Division of Population Science, Department of Medical Oncology, Kimmel Cancer Center, Thomas Jefferson University, Philadelphia, Pennsylvania, United States of America; 3 State Key Laboratory of Cancer Biology, Cell Engineering Research Centre and Department of Cell Biology, Fourth Military Medical University, Xi'an, China; University of Ottawa, Canada

## Abstract

**Background:**

Serum liver enzymes are frequently tested in clinics to aid disease diagnosis. Large observational studies indicated that these enzymes might predict cancer risk and mortality. However, no prospective study has reported on their relationships with the risk of HBV-related hepatocellular carcinoma (HCC).

**Methodology/Principal Findings:**

We evaluated the predictive values of four routinely tested liver enzymes (alanine aminotransferase [ALT], aspartate aminotransferase [AST], alkaline phosphatase [ALP], and gamma-glutamyltransferase [GGT]) in HCC risk in a prospectively enrolled clinical cohort of 588 Korean American HBV patients. For all four enzymes, the baseline level as well as the average and maximum levels during the first 1 or 2 years of follow-up were analyzed using multivariate Cox proportional hazards model. Patients were categorized into a normal or an elevated group based on the clinical cut-off of each enzyme. During a median follow-up of 7.5 years, 52 patients (incidence rate, 8.8%) developed HCC. The incidence rates were higher in the elevated groups for all four enzymes. The most significant finding was for GGT, with the highest incidence rate of 16.4% in the elevated group compared to 4.6% in the normal group (*P*<0.001). Compared to patients with normal baseline GGT, those with elevated GGT exhibited a significantly increased HCC risk with a hazards ratio (HR) of 2.60 (95% confidence interval [CI], 1.41–4.77, *P* = 0.002). Further analyses revealed a cumulative effect between baseline GGT and ALP (HR = 3.41, 95% CI 1.54–7.56, *P* = 0.003).

**Conclusions Significance:**

Serum GGT might predict HCC risk in HBV patients individually or jointly with other enzymes.

## Introduction

Worldwide, liver cancer is the second leading cause of cancer-related death in men and the sixth in women [1]. Hepatocellular carcinoma (HCC) accounts for >80% of liver cancer cases. Approximately 78% of HCC was attributable to hepatitis B virus (HBV) or hepatitis C virus (HCV) infection. Also, presence of cirrhosis from any cause markedly increases HCC risk [2]. The overall age-adjusted HCC incidence rate in the United States tripled between 1975 and 2005, partially accounted for by the increase of HCV infection and the influx of immigrants from HBV endemic regions. According to the World Health Organization, HCC has the second highest increase in overall death rate of all malignancies and its burden is expected to continue to increase in the next a few decades [3]. The five-year survival rate for HCC is <5% in all patients whereas >30% in patients diagnosed in early stages and receive surgery or liver transplantation [4]. These facts highlight the importance of clinical surveillance, risk prediction, targeted prevention, and early diagnosis in HCC management.

Serum liver enzymes such as alanine aminotransferase (ALT), aspartate aminotransferase (AST), alkaline phosphatase (ALP), and γ-glutamyltransferase (GGT), are tested routinely and automatically in current clinical settings. These enzymes are commonly elevated in patients with liver diseases and thus may reflect the status of liver injury [5]. Physicians generally use significant elevations of liver enzyme levels as complementary markers to aid the diagnosis of various diseases. For example, elevations of ALT and AST may indicate the presence of hepatocellular predominant disorders while elevations of ALP and GGT may implicate cholestatic predominant diseases [6]. Recent epidemiological studies have shown the associations between abnormally high liver enzyme levels and risks and mortalities of many diseases [7]–[10]. However, as yet, no population-based study has been reported on the associations of these enzymes and the risk of HCC in HBV patients, especially for baseline enzyme levels measured at the initial clinic visit of patients. In the current study, we sought to use a prospective approach to evaluate the effects of these four commonly tested serum liver enzymes on the long-term risk of developing HCC in a clinic-based Korean American HBV patient cohort. To the best of our knowledge, this is one of the first prospective studies that comprehensively evaluated these enzymes in HCC risk.

## Materials and Methods

### Study Population

The subjects in this study were identified from a clinic-based patient cohort. The patients in this cohort were consecutively enrolled from those who visited the Liver Disease Prevention Center at Thomas Jefferson University Hospital for the treatment of chronic HBV or HCV infection, and liver diseases such as cirrhosis, fibrosis, and/or HCC. There were no restrictions on age, gender, ethnicity, disease stage and etiology in enrollment. The enrollment started in 1988 and is ongoing. As of October 2010, the cohort included >2,600 patients. Because >90% of the patients in this cohort were of Korean ancestry, we restricted the study to Korean American patients to eliminate the confounding effect from patient ethnicity. For each patient, hepatitis viral infection status was clinically determined before or at enrollment. After the first visit, patients are followed at the center by 3–6 monthly intervals. During each visit, major variables such as liver function, complete blood count panel, kidney function, HBV DNA levels and AFP are measured. Abdominal imaging studies are carried out to detect early HCC. For this study, we included those patients who met all the following criteria: (1) patients had only HBV infection but no concomitant infection with HCV, HIV, or other viruses, in order to eliminate the confounding effects from disease etiology; (2) patients had all four serum liver enzymes (ALT, AST, ALP and GGT) measured simultaneously at study entry, in order to make the baseline analyses comparable; and (3) patients had a minimum follow-up time of 2 years from the study entry point and were not diagnosed with HCC within these 2 years of follow-up. This 2-year exclusion window will help minimize the confounding effects of those patients who actually developed HCC but not diagnosed at their initial clinical visits. This study was approved by the Institutional Review Board (IRB) at the Thomas Jefferson University. Because this study was based on data obtained from the review of archived medical charts, patient consent was waived by the IRB of the Office of Human Research in Thomas Jefferson University under an approved protocol including the approval for the request for waiver of authorization to collect protected health information.

### Data Collection

Demographic and clinical data were obtained from medical records to create a patient-level longitudinal database. Demographic variables collected for this study included age, gender, ethnicity, smoking status, alcohol consumption, and family history of cancer. Clinical variables were collected at study entry and follow-up visits with records of ALT, AST, ALP, and GGT values, as well as other routine clinical variables such as serum AFP, total bilirubin, direct bilirubin, cholesterol, triglycerides, count of white blood cells and platelets, prothrombin time, albumin, and ferritin, etc. Liver cirrhosis and HCC were determined through liver biopsy supplemented by imaging examinations, mainly magnetic resonance imaging (MRI) [11]. The endpoint of this study is HCC development. Time to HCC development was defined as the date from the study entry to the date of HCC diagnosis during follow-up, or the date of last follow-up if the patients were alive without HCC diagnosis at the time of this analysis. Patients free of HCC at death or at the end of last follow-up were censored for analysis.

### Determination of Cut-off Values of the Four Serum Liver Enzymes

The same clinically established protocol of liver function tests was used to measure the liver enzymes. Abnormal baseline enzyme levels were defined as the following: for ALT, the upper limit of normal (ULN) range is ≤40 U/L for males and ≤31 U/L for females; for AST, the ULN range is ≤37 U/L for males and ≤31 for females; for ALP, the ULN range is ≤117 U/L for males and females adults and 117–390 U/L for children (3–15 years old); for GGT, the ULN range is ≤51 U/L for males and ≤33 U/L for females [12], [13]. Since serum liver enzyme levels may fluctuate with the pathological states of patients, the use of a single time point of the enzyme level may result in unstable estimate. To address this caveat, we also analyzed the average and maximum enzyme levels during the first 1 year or 2 years of follow-up after study entry. The average or maximum liver enzyme levels were defined as the average or highest enzyme levels documented during the specified follow-up period of an individual.

### Statistical Analysis

Statistical analysis was performed using the SAS software version 9.2 (SAS Institute, Cary, NC). Continuous variables were expressed as mean with standard deviation (SD) and compared using the student’s *t* test or analysis of variance (ANOVA) test, where appropriate. Categorical variables were compared using the chi-square test or Fisher’s exact test, where appropriate. Count data (number of visit) was compared by using the Poisson regression analysis. The association between each liver enzyme and HCC risk was represented by hazards ratio (HR) and 95% confidence interval (CI) that were estimated using the Cox proportional hazards regression model, using univariate as well as multivariate analyses adjusting for age, gender, smoking status, alcohol consumption, cirrhosis and family history of cancer, where appropriate. Kaplan-Meier analysis was used to compare the cumulative risks of developing HCC in patients with different levels of serum liver enzymes, and log rank test was used to determine the statistical significance. We constructed receiving operating characteristics (ROC) curves and calculate the area under the curve (AUC), as well as positive predictive value (PPV) and negative predictive value (NPV) to evaluate the specificity and sensitivity of predicating HCC by using the combination of liver enzymes and epidemiological variables. A joint modeling approach implemented in the R package JM was used to conduct longitudinal analysis of GGT and HCC risk. Cumulative incidence of HCC by follow-up years was derived using Nelson-Aalen method [14]. The cumulative effects of GGT with other enzymes on HCC risk were analyzed by comparing patient with an elevated level of both enzymes to those with a normal level of both enzymes. All statistical tests were two-sided, and *P*<0.05 was considered the threshold of statistical significance.

## Results

### Characteristics of the Study Population

From the patients enrolled from 1988 through 2010, we identified 588 subjects that met the criteria described in Materials and Method. The general characteristics of these patients were summarized in **Table S1**. Among the 588 patients, 52 (8.8%) patients developed HCC within a median follow-up time of 7.5 years. The majority of patients are males (68.0%), younger than 50 years of old (76.9%), never smokers (69.0%), never drinkers (63.3%), non-cirrhotic (65.0%), and without a family history of cancer (66.7%). As expected, age and cirrhosis were both associated with a significantly increased risk of HCC (*P*<0.001). Males and ever smokers had a borderline significant increase in HCC risk (*P* value, 0.091 and 0.066, respectively). The association between HCC risk and alcohol consumption or family history of cancer was not significant (**Table S1**). The distributions of patient characteristics in relation to the four liver enzyme levels were listed in **Table 1**. A significantly higher percentage of cirrhotic patients had an elevated level for all the four tested enzymes (*P*<0.001 for all four enzymes). In addition, a significantly elevated enzyme level was observed in males and ever smokers for ALT, in males and ever drinkers for ALP, and in older patients, ever smokers and ever drinkers for GGT. Higher HCC incidences were observed in patients with elevated levels for all four enzymes, especially for GGT which was associated with the highest HCC incidence of 16.4% in the elevated group compared to 4.6% in the normal group (*P*<0.001). There were no significant differences between the normal and elevated groups for all four enzymes with respect to age at HCC diagnosis and total follow-up years. For all four enzymes, patients in the elevated group had a significantly higher number of enzyme measurements compared to those in the normal group (*P*<0.001 for all four enzymes), which is consistent with clinical practices that the patients with higher enzyme levels might have more severe diseases and thus had a higher number of clinical visits.

**Table 1:** Characteristics of the study population by serum liver enzymes activity status at baseline.

Variables	ALT^1^			AST^2^			ALP^3^			GGT^4^		
	Normal (n = 238)	Elevated (n = 350)	*P* value	Normal (n = 302)	Elevated (n = 286)	*P* value	Normal (n = 494)	Elevated (n = 94)	*P* value	Normal (n = 374)	Elevated (n = 214)	*P* value
Gender												
Female	87	101		98	90		169	19		128	60	
Male	151	249	**0.049**	204	196)	0.799	325	75	**0.008**	246	154	0.122
Age												
≤39	94	158		139	113		215	37		181	71	
40–49	85	115		99	101		172	28		123	77	
50–59	43	59		49	53		81	21		49	53	
≥60	16	18	0.547	15	19	0.408	26	8	0.266	21	13	**<0.001**
Smoking status												
Never	177	229		213	193		348	58		276	130	
Ever	61	121	**0.021**	89	93	0.424	146	36	0.093	98	84	**0.001**
Alcohol consumption												
Never	160	212		196	176		323	49		251	121	
Ever	78	138	0.100	106	110	0.398	171	45	**0.015**	123	93	**0.011**
Cirrhosis												
No	175	207		227	155		342	40		271	111	
Yes	63	143	**<0.001**	75	131	**<0.001**	152	54	**<0.001**	103	103	**<0.001**
Family cancer												
No	162	230		199	193		328	64		246	146	
Yes	76	120	0.552	103	93	0.683	166	30	0.750	128	68	0.544
	17 (7.1)	35 (10.0)	0.217	19 (6.3)	33 (11.5)	**0.026**	37 (7.5)	15 (15.6)	**0.032**	17 (4.6)	35 (16.4)	**<0.001**

Notes: ^1^The cutoff values for ALT are: Normal, ALT ≤40.0 U/L for male or ≤31.0 U/L for female; Elevated, ALT >40.0 U/L for male or >31.0 U/L for female;

2 the cutoff values for AST are: Normal, AST ≤37.0 U/L for male or ≤31.0 U/L for female; Elevated, AST >37.0 U/L for male or >31.0 U/L for female;

3 the cutoff values for ALP are: Normal, ALP≤117.0 U/L for all patients; Elevated, ALP>117.0 U/L for adults and 117–390 for children (3–15 years);

4 the cutoff values for GGT are: Normal, GGT ≤51.0 U/L for male or GGT ≤33.0 U/L for female; Elevated, GGT >51.0 U/L for male or >33.0 U/L for female.

### The Association of Liver Enzymes with HCC Risk in HBV Patients

We analyzed the associations between each of the four liver enzymes and HCC risk using univariate and multivariate Cox proportional hazards regression analyses (**Table 2**). Because cirrhosis is one of the strongest risk factors of HCC and might also be related to liver enzyme levels, we conducted the multivariate analyses with (the middle column of multivariate analysis of Table 2) and without (the last column of Table 2) the adjustment for cirrhosis. In order to minimize the variations resulting from the use of a single time point, we also analyzed, in addition to the baseline level at study entry, the average and maximum levels within the first one year **(** **Table 2** **)** and two years **(Table S3)** of follow-up. As shown in **Table 2**, ALT was not associated with an altered HCC risk in both univariate and multivariate analyses. Since there was no universally agreed definition of abnormal ALT cut-off in the clinical setting, we conducted the analyses by different ALT cut-off values used in clinics, and did not find any significant result in the univariate and multivariate analyses for any of these cut-off values (**Table S2**). Baseline AST was associated with an increased HCC risk in the univariate analysis (HR = 1.93, 95% CI 1.09–3.41, *P* = 0.023) and the multivariate analysis without adjusting cirrhosis (HR = 2.04, 95% CI 1.14–3.67, *P* = 0.017). However, the significant association disappeared when the analysis was adjusted for cirrhosis (HR = 1.35, 95% CI 0.74–2.49, *P* = 0.331). Similar results were noticed for ALP, for which all five tested enzyme levels were associated with an increased HCC risk in the univariate analysis and multivariate analysis without adjusting cirrhosis, which disappeared when cirrhosis was adjusted in the multivariate analysis. These results indicated that the increased HCC risk conferred by elevated AST and ALP levels were likely mediated by cirrhosis. In comparison, GGT exhibited the most significant association HCC risk. For example, abnormal baseline GGT level conferred a significant HCC risk with an HR of 3.91 (95% CI 2.18–7.00, *P*<0.001) for univariate analysis and 2.86 (95% CI 1.55–5.26, *P* = 0.001) for the multivariate analysis without adjusting cirrhosis. When cirrhosis was included in the multivariate analysis, the association, although attenuated, remained statistically significant with an HR of 2.60 (95% CI 1.41–4.77, *P* = 0.002) (**Table 2**). Similar results were noticed for average and maximum GGT levels in one and two years of follow-up (**Table S2 and Table S3**). These data strongly suggested that serum GGT was an independent prospective predictor of HCC risk in HBV patients. We further categorized GGT levels to normal (≤ ULN), elevated (1–2x ULN) and highly elevated (>2x ULN) groups to evaluate if the effects observed for GGT were dose-dependent. In the univariate analysis, a highly significant dose response was observed (*P* trend<0.001 for all analyses, **Table S4**). The effect, although attenuated, remained significant after adjusting for the major variables including cirrhosis (*P* trend ranged from 0.002 to 0.043 for the five different GGT measurements, **Table S4**). This analysis further confirmed serum GGT as a robust independent HCC predictor. We further conducted stratified analysis of GGT by demographic variables and cirrhosis. In the multivariate analysis adjusting all variables, the GGT-HCC association remained significant in males (*P* = 0.006), older patients (*P* = 0.003), never (*P* = 0.021) and ever smokers (*P* = 0.011), never (*P* = 0.036) and ever drinkers (*P* = 0.006), cirrhotic patients (*P* = 0.003), and patients without a family history of cancer (*P* = 0.014) (**Table S5**). The AUC was 0.84 for ROC constructed incorporating age, gender, smoking status, alcohol consumption, cirrhosis, family history of cancer, and GGT. The NPV was 99.15 and the PPV was 20.67. The results of our ROC analysis had a low false-negative rate whereas a high rate of false-positive. Therefore, additional approaches are necessary to further identify patients who are most likely to develop HCC. We further conducted a longitudinal analysis of GGT and the risk of developing HCC in HBV patients. Similarly, we found a significant association between log (GGT) and HCC risk with HR of 2.03 (95% CI 1.68–2.45, *P* = 0.0002) (Data not shown).

**Table 2:** The association of serum liver enzyme levels within 1 year of follow-up and HCC risk in HBV-infected patients.

Enzymes	Serum enzyme level status^1^	Case/total	Univariate		Multivariate-adjusted^2^		Multivariate-adjusted^3^	
			HR (95% CI)	*P* value	HR (95% CI)	*P* value	HR (95% CI)	*P* value
ALT	By baseline							
	Normal	17/238	1		1		1	
	Elevated	35/350	1.33(0.74–2.38)	0.342	1.21(0.65–2.28)	0.548	1.68(0.90–3.12)	0.104
	By average in first 1 year of follow-up							
	Normal	14/234	1		1		1	
	Elevated	38/354	1.58(0.85–2.93)	0.145	1.16(0.60–2.26)	0.661	1.56(0.82–2.97)	0.18
	By maximum in first 1 year of follow-up							
	Normal	12/190	1		1		1	
	Elevated	40/398	1.56(0.82–2.99)	0.177	1.04(0.51–2.13)	0.914	1.55(0.78–3.07)	0.207
AST	By baseline							
	Normal	19/302	1		1		1	
	Elevated	33/286	**1.93(1.09–3.41)**	**0.023**	1.35(0.74–2.49)	0.331	**2.04(1.14–3.67)**	**0.017**
	By average in first 1 year of follow-up							
	Normal	18/294	1		1		1	
	Elevated	34/294	1.61(0.91–2.87)	0.104	0.92(0.49–1.75)	0.805	1.41(0.77–2.58)	0.268
	By maximum in first 1 year of follow-up							
	Normal	15/257	1		1		1	
	Elevated	37/331	**1.84(1.00–3.36)**	**0.049**	1.06(0.54–2.06)	0.872	1.65(0.88–3.09)	0.122
ALP	By baseline							
	Normal	37/494	1		1		1	
	Elevated	15/94	**3.22(1.74–5.97)**	**<0.001**	1.60(0.84–3.04)	0.153	**2.23(1.19–4.19)**	**0.013**
	By average in first 1 year of follow-up							
	Normal	38/505	1		1		1	
	Elevated	14/83	**3.61(1.93–6.78)**	**<0.001**	1.75(0.88–3.48)	0.11	**2.42(1.25–4.68)**	**0.009**
	By maximum in first 1 year of follow-up							
	Normal	34/454	1		1		1	
	Elevated	18/134	**2.62(1.46–4.73)**	**0.001**	1.58(0.85–2.93)	0.149	**2.10(1.15–3.84)**	**0.016**
GGT	By baseline							
	Normal	17/374	1		1		1	
	Elevated	35/214	**3.91(2.18–7.00)**	**<0.001**	**2.60(1.41–4.77)**	**0.002**	**2.86(1.55–5.26)**	**0.001**
	By average in first 1 year of follow-up							
	Normal	16/374	1		1		1	
	Elevated	36/214	**3.68(2.04–6.65)**	**<0.001**	**2.04(1.07–3.90)**	**0.031**	**2.47(1.31–4.66)**	**0.005**
	By maximum in first 1 year of follow-up							
	Normal	13/337	1		1		1	
	Elevated	39/251	**3.63(1.93–6.82)**	**<0.001**	1.94(0.97–3.88)	0.059	**2.33(1.19–4.57)**	**0.014**

Notes: ^1^The cutoff values for ALT are: Normal, ALT ≤40.0 U/L for male or ≤31.0 U/L for female; Elevated, ALT >40.0 U/L for male or >31.0 U/L for female; the cutoff values for AST are: Normal, AST ≤37.0 U/L for male or ≤31.0 U/L for female; Elevated, AST >37.0 U/L for male or >31.0 U/L for female; the cutoff values for ALP are: Normal, ALP≤117.0 U/L for all patients; Elevated, ALP>117.0 U/L for adults and 117–390 for children (3–15 years); the cutoff values for GGT are: Normal, GGT ≤51.0 U/L for male or GGT ≤33.0 U/L for female; Elevated, GGT >51.0 U/L for male or >33.0 U/L for female.

2 HR adjusted for gender, age, smoking status, alcohol consumption, cirrhosis, and family cancer.

3 HR adjusted for gender, age, smoking status, alcohol consumption, and family cancer.

### Cumulative Risk of HCC by Baseline Serum Liver Enzymes

**Figure 1:**
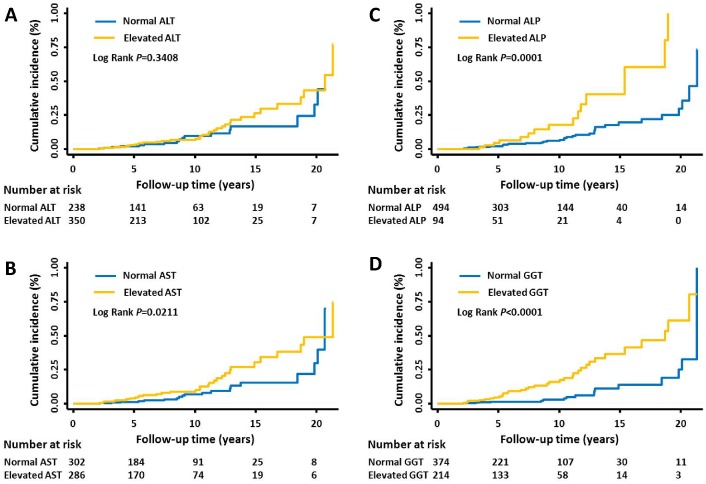
Cumulative incidence of HCC by the clinical cut-off values of baseline enzyme levels. HCC incidence by the clinical cut-off of baseline (A) ALT, (B) AST, (C) ALP, and (D) GGT.

The cumulative incidence of HCC in our study population by baseline serum liver enzymes during the complete follow-up period (1988–2010) was shown in **Figure 1**. An increasing trend of cumulative HCC incidences over the follow-up period was observed for all four enzymes. The log rank *P* value was 0.341, 0.021, 0.0001 and <0.001 for ALT, AST, ALP, and GGT, respectively (**Figure 1**). In line with the results of Table 2, GGT exhibited the best discriminative capacity. The detailed cumulative incidence of HCC in the follow-up of 3, 6, 9, 12, 15, and >18 years were shown in **Table S6**. Consistently, GGT exhibited the strongest discriminative capacity. For instance, for patients with >18 years of follow-up, the cumulative incidence in those with normal vs. elevated enzyme levels was 44.0 vs. 77.3 for ALT, 69.9 vs. 74.5 for AST, 73.2 vs. 80.2 for ALP, whereas 32.7 vs. 80.6 for GGT. Patients with the normal vs. elevated levels of GGT had the largest difference in HCC incidence for all the follow-up periods examined (**Table S6**).

### Combined Effects of GGT with Other Liver Enzymes on HCC Risk

GGT was commonly tested in the hepatic panel together with other liver enzymes in clinical settings [6]. The joint analysis of GGT with other enzymes may yield additional information regarding disease risk and diagnosis. For example, elevated GGT combined with elevated ALP usually points to hepatobiliary injury, which distinguishes from ALP elevation alone resulting from bone diseases [15]. We further combined GGT with ALT, AST or ALP to determine if the combined evaluation could improve the predictive power compared to GGT alone. As shown in **Table 3**, the combined analysis of GGT with ALP markedly increased HCC risk in patients with an elevated level of both enzymes compared to those with a normal value for both enzymes. For example, in baseline analysis, the unadjusted HR increased from 3.91 (2.18–7.00, *P*<0.001) in GGT alone (**Table 2**) to 8.06 (3.87–16.8, *P*<0.001) in the combined use of GGT with ALP (**Table 3**). The multivariate adjusted HR increased from 2.60 (1.41–4.77, *P* = 0.002, adjusting all variables) and 2.86 (1.55–5.26, *P* = 0.001, without adjusting cirrhosis) in GGT alone to 3.41 (1.54–7.56, *P* = 0.003) and 4.47 (2.05–9.76, *P*<0.001) in the combination of GGT with ALP, respectively. Similar levels of increased effects were observed for the use of average or maximum GGT level (**Table 3**). In comparison, the combinations of GGT with ALT or AST did not exhibit a significant association with HCC risk in the multivariate analysis adjusting for cirrhosis (**Table 3**).

**Table 3:** Combined effects of GGT with other liver enzymes on HCC risk.

Enzymes combination	Serum enzyme level status^1^	Case/total	Univariate		Multivariate-adjusted^2^		Multivariate-adjusted^3^	
			HR (95%CI)	P value	HR (95%CI)	P value	HR (95%CI)	P value
GGT + ALT	By baseline							
	Normal	10/211	1.00		1.00		1.00	
	Elevated	28/187	**3.36(1.62–6.95)**	**0.001**	**2.18(1.00–4.75)**	**0.049**	**3.05(1.40–6.63)**	**0.005**
	By average in first 1 year of follow-up							
	Normal	7/207	1.00		1.00		1.00	
	Elevated	29/187	**4.11(1.80–9.40)**	**0.001**	1.89(0.75–4.74)	0.177	**2.69(1.11–6.50)**	**0.028**
	By maximum in first 1 year of follow-up							
	Normal	6/173	1.00		1.00		1.00	
	Elevated	33/234	**3.83(1.60–9.17)**	**0.003**	1.74(0.65–4.63)	0.271	**2.54(1.00–6.43)**	**0.049**
	By average in first 2 year of follow-up							
	Normal	6/203	1.00		1.00		1.00	
	Elevated	26/177	**4.45(1.82–10.9)**	**0.001**	2.02(0.75–5.45)	0.165	**2.98(1.14–7.79)**	**0.026**
	By maximum in first 2 year of follow-up							
	Normal	6/149	1.00		1.00		1.00	
	Elevated	33/242	**3.29(1.37–7.89)**	**0.008**	1.47(0.54–4.01)	0.448	2.31(0.91–5.86)	0.078
GGT + AST	By baseline							
	Normal	10/263	1.00		1.00		1.00	
	Elevated	26/175	**4.63(2.22–9.69)**	**<0.001**	**2.80(1.27–6.15)**	**0.010**	**3.89(1.79–8.47)**	**0.001**
	By average in first 1 year of follow-up							
	Normal	10/258	1.00		1.00		1.00	
	Elevated	28/178	**3.53(1.71–7.29)**	**0.001**	1.59(0.69–3.68)	0.280	**2.48(1.13–5.43)**	**0.023**
	By maximum in first 1 year of follow-up							
	Normal	8/225	1.00		1.00		1.00	
	Elevated	32/219	**3.70(1.70–8.07)**	**0.001**	1.71(0.70–4.16)	0.240	**2.51(1.09–5.78)**	**0.031**
	By average in first 2 year of follow-up							
	Normal	10/263	1.00		1.00		1.00	
	Elevated	27/173	**3.66(1.77–7.60)**	**<0.001**	1.55(0.68–3.50)	0.297	**2.63(1.20–5.76)**	**0.015**
	By maximum in first 2 year of follow-up							
	Normal	8/200	1.00		1.00		1.00	
	Elevated	33/232	**3.35(1.54–7.27)**	**0.002**	1.48(0.60–3.65)	0.392	2.31(1.00–5.33)	0.050
GGT + ALP	By baseline							
	Normal	17/339	1.00		1.00		1.00	
	Elevated	15/59	**8.06(3.87–16.8)**	**<0.001**	**3.41(1.54–7.56)**	**0.003**	**4.47(2.05–9.76)**	**<0.001**
	By average in first 1 year of follow-up							
	Normal	15/339	1.00		1.00		1.00	
	Elevated	13/48	**9.07(4.12–20.0)**	**<0.001**	**3.17(1.29–7.81)**	**0.012**	**4.59(1.94–10.9)**	**0.001**
	By maximum in first 1 year of follow-up							
	Normal	12/286	1.00		1.00		1.00	
	Elevated	17/83	**6.46(2.95–14.1)**	**<0.001**	**2.59(1.06–6.31)**	**0.036**	**3.56(1.53–8.29)**	**0.003**
	By average in first 2 year of follow-up							
	Normal	16/350	1.00		1.00		1.00	
	Elevated	13/42	**9.24(4.27–20.0)**	**<0.001**	**3.51(1.46–8.43)**	**0.005**	**4.60(1.98–10.7)**	**<0.001**
	By maximum in first 2 year of follow-up							
	Normal	12/272	1.00		1.00		1.00	
	Elevated	17/92	**5.52(2.52–12.1)**	**<0.001**	2.16(0.87–5.35)	0.097	**3.15(1.35–7.31)**	**0.008**

Notes: ^1^The cutoff values for ALT are: Normal, ALT ≤40.0 U/L for male or ≤31.0 U/L for female; Elevated, ALT >40.0 U/L for male or >31.0 U/L for female; the cutoff values for AST are: Normal, AST ≤37.0 U/L for male or ≤31.0 U/L for female; Elevated, AST >37.0 U/L for male or >31.0 U/L for female; the cutoff values for ALP are: Normal, ALP≤117.0 U/L for all patients; Elevated, ALP>117.0 U/L for all patients; the cutoff values for GGT are: Normal, GGT ≤51.0 U/L for male or GGT ≤33.0 U/L for female; Elevated, GGT >51.0 U/L for male or >33.0 U/L for female. Normal, both enzymes were at normal range; Elevated, both enzymes were at elevated levels.

2 HR adjusted for gender, age, smoking status, alcohol consumption, cirrhosis, and family cancer.

3 HR adjusted for gender, age, smoking status, alcohol consumption, and family cancer.

## Discussion

We extensively evaluated the associations between four common liver enzymes (ALT, AST, ALP and GGT) that are routinely tested in the clinical setting, and the risk of developing HCC in a prospectively enrolled cohort of HBV patients. We found that the clinically significant elevation of serum GGT level, either at baseline or determined using the average or maximum level of one or two years of follow-up, independently predicted the risk of developing HCC in HBV patients. Moreover, we demonstrated that this effect was dose-dependent and markedly increased when combined with the use of elevated level of ALP but not ALT or AST.

There are two major types of serum liver enzyme level changes commonly encountered in clinical practice: hepatocellular predominance with elevated ALT and AST, and cholestatic predominance with elevated ALP and GGT [6]. Serum ALT and AST are released from damaged hepatocytes into blood and their activities have been widely recognized as effective tools to detect liver diseases [16]–[20]. Actually, ALT is the most extensively investigated serum enzyme and elevated ALT has been associated with the mortality of various liver diseases [18], [21]. In our study, we did not observe a significant association between ALT and HCC risk in either univariate or multivariate analysis, suggesting the inability of ALT as a prospective predictor of HCC risk in HBV patients. This was in line with the findings from several recent studies reporting that biomarkers such as high HBV DNA load or HBeAg seroconversion predict the increased risk or mortality of various liver diseases only in patients with normal serum ALT levels [10], [22]–[24], which further indicated the complexity of ALT as an HCC risk factor. In our study, AST exhibited a significant association with HCC risk in the univariate analysis, which disappeared after multivariate analysis adjusting all the major variables including cirrhosis. Similar observations were also noticed for ALP (**Table 2**). These data indicated that the associations observed for AST and ALP could be potentially mediated by the presence of liver cirrhosis. Previous observations have shown a correlation between AST/ALT ratio and presence of liver cirrhosis whereas the association between AST or ALP alone with cirrhosis were not extensively studied [25]. Additional independent studies are needed to further confirm the roles of these enzymes in predicting HCC risk in the context of cirrhosis.

In our study, GGT was the only enzyme that predicts the risk of HCC independent of other major demographic variables and liver cirrhosis. This result was consistent with several previous studies showing that GGT was a better predictor than ALT for some liver-specific diseases in both HBV patients and the general population [13], [26]. In addition to an indicator for hepatobiliary disorders, elevation of serum GGT has been reported in a variety of clinical conditions, including pancreatic disease, myocardial infarction, renal failure, diabetes, and alcoholism [5], [27], [28]. In addition, significant associations of elevated GGT with the risk of several cancers have been reported [7], [26], [29]_ENREF_10. However, very few studies have examined the prospective predictive role of GGT in the risk of HBV-related HCC. As yet, there is only one study reporting such an association but the population only included men and GGT was not the major finding of that study [26]. To the best of our knowledge, the present study is the first one to report a strong correlation between elevated serum GGT and the risk of HBV-related HCC. Based on our large and prospective clinical cohort population, the significant GGT-HCC association was independent of other major demographic and clinical variables including age, gender, smoking status, alcohol consumption, cirrhosis, and family history of cancer. In this study, we did not observe a prominent association in females, younger patients, and patients with a family history of cancer, which were possibly accounted for by the small number of patients who developed HCC in these strata (**Table S5**). Moreover, only a small number (8 out of 52) of HBV patients who developed HCC without cirrhosis and the multivariate analysis could not estimate a reliable association between GGT and HCC risk in those non-cirrhotic patients (**TableS 5**). However, the GGT-HCC association remained significant in cirrhotic patients after adjusting for all other variables. This stratified analysis by cirrhosis echoed the finding of the main effects analyses, that is, unlike ALT, AST, and ALP, GGT was the only enzyme that exhibited a significant association with HCC risk independent of other major variables. These results suggested that the effects conferred by elevated serum GGT on HCC risk could not be overshadowed by cirrhosis, the strongest risk factor in developing HCC under any causes.

Serum GGT level has been investigated and developed as a liver function test for several decades [30], [31]; however, the molecular mechanisms of GGT in HCC development still remains unclear. Experimental studies have indicated that serum GGT was not simply derive from the enzyme release from damaged liver cells. Instead, it was possible that GGT expressed on the cell surface is released into circulation, which is facilitated by bile acids acting on the cell membrane [28]. This mechanism is different from ALT and AST that are released directly from injured liver cells [6]. Since chronic HBV infection is commonly accompanied by severe injuries of hepatocytes, this mechanism could possibly explain the fact that GGT was a better predicator than ALT or AST in our population of HBV patients. Moreover, it has been suggested that the role of GGT in tumorigenesis might be mediated by the functions of the oxidative stress pathways in cellular response [32]. GGT is a membrane-bound enzyme that catalyzes the degradation of extracellular glutathione (GSH) and allows the component amino acids to be available for intracellular GSH re-synthesis [28]. GSH is essential in the protection of cells from damages induced by oxidants generated during normal metabolism. There is extensive evidence that GGT and GSH can cooperatively generate free radicals and thus lead to lipid peroxidation. Since lipid peroxidation and other metabolisms were significantly implicated in the tumorigenesis of many malignancies including HCC, this might also partially explain the GGT-HCC association observed in our study [33], [34]_ENREF_37. Nonetheless, the in-depth molecular and cellular mechanisms underlying the findings of this study warrant further physiological characterizations.

In addition to viral infections and cirrhosis, environmental factors such as smoking and alcohol intake are well known risk factors of HCC development. In our study, we noticed that ever smoking conferred a borderline increase in HCC risk which was consistent with previous studies [35]. However, we did not observe a significant association between ever drinking and HCC risk in our study (**Table S1**). Many studies have suggested that there was a threshold of alcohol intake in relation to HCC risk. Moreover, some studies reported that light drinking might have a protective effect for HCC development [36], [37]. Unfortunately, we do not have the data on the intensities of smoking and drinking in this study population, which limited our further analyses of their interactions with GGT on HCC development.

We noticed that the average age (about 50 years old) at diagnosis of HCC patients is relatively young compared to patients in the Surveillance Epidemiology and End Results (SEER) database. The majority of HCC patients in the SEER database had HCV-related HCC. It is known that the age of HCC diagnosis is younger in HBV-HCC compared to HCV- HCC patients by one decade in average, because of the different mode/time of viral transmission. In the endemic region, HBV infection takes place during neonatal period and/or infancy in the majority of cases while HCV infection occurs later, e.g., from blood transfusion. This could partly explain the relatively younger age for HBV-HCC in our study. In addition, in our study, most HCC patients (44/52) were males who are usually younger than female HCC patients.

Our study has several strengths. To our knowledge, this is one of the largest clinic-based cohorts that have been completely enrolled in a single institute. The unique and highly homogeneous Korean American HBV patient populations eliminated the confounding effects from ethnicity. The highly homogenous of HCC etiology with uniformly HBV genotype C infection is another important strength. The majority of the patients in this study were infected with HBV at birth or childhood and immigrated to the United State at young age, especially those who developed HCC, making our population an ideal resource to study the long-term outcome of HBV infection at the population level. More importantly, our study used a prospective design with the baseline data collected many years ahead of HCC diagnosis, which circumvented the reverse-causation issue inherent in most retrospective case-control epidemiological studies. All of the included patients had complete data of the four liver enzymes measured simultaneously at study entry, making the analyses completely comparable. The strict restriction of patients to those with a ≥2 years of follow-up and exclusion of those who developed HCC within this 2-year window further significantly reduced the chance of false positive findings. Finally, we extensively analyzed the data by different liver enzymes levels during different periods of follow-up, instead of only using the baseline level. Results of the analyses of the average and maximum enzymes levels during one or two years of follow-up were highly consistent with the baseline findings. Despite these strong strengths, our study also had limitations. First, the generalizability of our findings to other ethnic groups as well as to patients with other HCC etiologies such as HCV infection remained to be assessed. Second, the total number of 2,600 was the entire number of patients seen at the center over a span of 23 years. While the majority of them (>90%) were HBV patients, there were others including patients with other conditions such as HCV, Nonalcoholic steatohepatitis (NASH), alcoholic liver disease, hemochromatosis, drug-induced hepatitis, primary biliary cirrhosis, autoimmune hepatitis, and etc. During the 23 years’ period, some patients returned to their physicians and some did not return or moved away. Moreover, some had irregular visits in years and also many died. The 588 patients were those who were on regular follow-up with available lab test data on the four liver enzymes during each visit. This high attrition rate of our population is another limitation of generalization to common population. Third, the lack of accurate alcohol consumption data is a major limitation, as exemplified by the fact that ever drinkers did not exhibit elevated AST levels. Due to the incomplete data on smoking and drinking intensity, we are not able to analyze the cumulative and interaction effects between GGT and smoking or drinking intensity. Fourth, we do not have the complete data of some other clinical variables determined at the same time when all the four enzymes were measured. Another limitation of our study is the lack of data on the lag time between diagnosis of HBV infection and enzyme measurement. Future prospective studies are warranted to analyze the influence of this factor on HCC incidence in HBV patients.

In summary, based on a large prospectively enrolled clinic-based HBV patient cohort, we found that the elevation of serum GGT level could potentially be used as a prospective biomarker of the long-term risk of developing HCC in HBV patients. If validated, GGT may be combined with other major HCC risk factors to build a HCC risk assessment model that can be applied in the clinical settings.

## Supporting Information

Table S1 **The associations of demographic variables and the risk of developing HCC in HBV patients.**
(DOCX)

Table S2 **The association of ALT and HCC risk by different cutoff values.**
(DOCX)

Table S3 **The association of serum liver enzyme levels within 2 years of follow-up and HCC risk in HBV-infected patients.**
(DOCX)

Table S4 **The association of serum GGT levels and HCC risk in HBV-infected patients with dose-dependent manner.**
(DOCX)

Table S5 **The association of baseline GGT levels and the risk of HCC stratified by demographic variables.**
(DOCX)

Table S6 **Cumulative incidence of HCC by serum liver enzymes at baseline levels and different years of follow-up.**
(DOCX)
