# Supplementary material for: Comprehensive Analysis of Common Serum Liver Enzymes as Prospective Predictors of Hepatocellular Carcinoma in HBV Patients
Source: PLoS One. 2012 Oct 24;7(10):e47687. doi: 10.1371/journal.pone.0047687 (PMC3480412; doi:10.1371/journal.pone.0047687)
Supplement: Table S1 — The associations of demographic variables and the risk of developing HCC in HBV patients. (DOCX) [file pone.0047687.s001.docx]

| **Supplementary Table S1. The associations of demographic variables and the risk of developing HCC in HBV patients** | | | | |
| --- | --- | --- | --- | --- |
| Variables | Number (%) of total patients (n=588) | Number (%) of HCC patients (n=52) | HR (95% CI)^1^ | *P* value |
| gender |  |  |  |  |
| Female | 188 (32.0) | 8 (15.4) | 1.00 |  |
| Male | 400 (68.0) | 44 (84.6) | 2.09(0.89-4.93) | 0.091 |
| Age |  |  |  |  |
| ≤ 39 | 252 (42.9) | 9 (17.3) | 1.00 |  |
| 40-49 | 200 (34.0) | 16 (30.8) | **3.20(1.38-7.38)** | **0.007** |
| 50-59 | 102 (17.3) | 21 (40.4) | **12.3(5.14-29.4)** | **< 0.001** |
| ≥ 60 | 34 (5.8) | 6 (11.5) | **19.7(6.11-63.2)** | **< 0.001** |
| Smoking status |  |  |  |  |
| Never | 406 (69.0) | 28 (53.8) | 1.00 |  |
| Ever | 182 (31.0) | 24 (46.2) | 2.05(0.95-4.41) | 0.066 |
| Alcohol consumption |  |  |  |  |
| Never | 372 (63.3) | 28 (53.8) | 1.00 |  |
| Ever | 216 (36.7) | 24 (46.2) | 0.74(0.35-1.56) | 0.427 |
| Cirrhosis |  |  |  |  |
| No | 382 (65.0) | 8 (15.4) | 1.00 |  |
| Yes | 206 (35.0) | 44 (84.6) | **5.52(2.54-12.0)** | **< 0.001** |
| Family history of cancer |  |  |  |  |
| No | 392 (66.7) | 32 (61.5) | 1.00 |  |
| Yes | 196 (32.3) | 20 (38.5) | 1.10(0.61-2.00) | 0.742 |
| Note: ^1^Adjusted for gender, age, smoking status, drinking status, cirrhosis, and family history of cancer, where appropriately. | | | | |
