# Supplementary material for: Comprehensive Analysis of Common Serum Liver Enzymes as Prospective Predictors of Hepatocellular Carcinoma in HBV Patients
Source: PLoS One. 2012 Oct 24;7(10):e47687. doi: 10.1371/journal.pone.0047687 (PMC3480412; doi:10.1371/journal.pone.0047687)
Supplement: Table S2 — The association of ALT and HCC risk by different cutoff values. (DOCX) [file pone.0047687.s002.docx]

| **Supplementary Table S2. The association of ALT and HCC risk by different cutoff values** | | | | | | | | | | |
| --- | --- | --- | --- | --- | --- | --- | --- | --- | --- | --- |
| Serum enzyme level status | Number of patients (n=588) | Number of cases (n=52) | Unadjusted | |  | Multivariate-adjusted^1^ | |  | Multivariate-adjusted^2^ | |
|  |  |  | HR (95% CI) | *P* value |  | HR (95% CI) | *P* value |  | HR (95% CI) | *P* value |
| **ALT cutoff by ≤ 33 U/L for males and ≤ 25 U/L for females** | | | | | | | | | | |
| By baseline |  |  |  |  |  |  |  |  |  |  |
| Normal | 186 | 12 |  |  |  |  |  |  | 1.00 |  |
| Elevated | 402 | 40 | 1.48(0.78-2.84) | 0.233 |  | 0.98(0.48-2.00) | 0.959 |  | 1.39(0.70-2.77) | 0.349 |
| By average in first 1 year of follow-up | |  |  |  |  |  |  |  |  |  |
| Normal | 173 | 9 |  |  |  |  |  |  |  |  |
| Elevated | 415 | 43 | 1.92(0.93-3.96) | 0.076 |  | 1.26(0.57-2.78) | 0.569 |  | 1.90(0.89-4.07) | 0.097 |
| By maximum in first 1 year of follow-up | | |  |  |  |  |  |  |  |  |
| Normal | 146 | 9 |  |  |  |  |  |  |  |  |
| Elevated | 442 | 43 | 1.56(0.76-3.22) | 0.225 |  | 0.98(0.44-2.20) | 0.961 |  | 1.31(0.61-2.83) | 0.491 |
| By average in first 2 years of follow-up | | |  |  |  |  |  |  |  |  |
| Normal | 177 | 10 |  |  |  |  |  |  |  |  |
| Elevated | 411 | 42 | 1.75(0.87-3.49) | 0.115 |  | 1.23(0.58-2.61) | 0.597 |  | 1.79(0.86-3.73) | 0.117 |
| By maximum in first 2 years of follow-up | | |  |  |  |  |  |  |  |  |
| Normal | 131 | 9 |  |  |  |  |  |  |  |  |
| Elevated | 457 | 43 | 1.41(0.68-2.91) | 0.350 |  | 0.87(0.38-1.97) | 0.735 |  | 1.21(0.56-2.62) | 0.627 |
|  |  |  |  |  |  |  |  |  |  |  |
| **ALT cutoff by ≤ 30 U/L for males and ≤ 19 U/L for females** | | | | | | | | | | |
| By baseline |  |  |  |  |  |  |  |  |  |  |
| Normal | 137 | 8 |  |  |  |  |  |  |  |  |
| Elevated | 451 | 44 | 1.87(0.88-3.98) | 0.106 |  | 0.88(0.40-1.97) | 0.759 |  | 1.47(0.67-3.24) | 0.336 |
| By average in first 1 year of follow-up | |  |  |  |  |  |  |  |  |  |
| Normal | 127 | 6 |  |  |  |  |  |  |  |  |
| Elevated | 461 | 46 | 2.11(0.90-4.95) | 0.087 |  | 0.95(0.39-2.34) | 0.914 |  | 1.42(0.58-3.47) | 0.445 |
| By maximum in first 1 year of follow-up | | |  |  |  |  |  |  |  |  |
| Normal | 106 | 6 |  |  |  |  |  |  |  |  |
| Elevated | 482 | 46 | 1.97(0.84-4.63) | 0.121 |  | 0.77(0.31-1.92) | 0.577 |  | 1.28(0.52-3.14) | 0.590 |
| By average in first 2 years of follow-up | | |  |  |  |  |  |  |  |  |
| Normal | 133 | 8 |  |  |  |  |  |  |  |  |
| Elevated | 455 | 44 | 1.62(0.76-3.46) | 0.212 |  | 1.30(0.57-2.96) | 0.537 |  | 1.51(0.66-3.42) | 0.327 |
| By maximum in first 2 years of follow-up | | |  |  |  |  |  |  |  |  |
| Normal | 93 | 6 |  |  |  |  |  |  |  |  |
| Elevated | 495 | 46 | 1.63(0.69-3.83) | 0.262 |  | 0.75(0.30-1.86) | 0.531 |  | 1.19(0.48-2.93) | 0.704 |
| Notes: ^1^HR adjusted for gender, age, smoking status, drinking status, cirrhosis, and family cancer. ^2^HR adjusted for gender, age, smoking status, drinking status, and family cancer. | | | | | | | | | | |
