# Supplementary material for: Comprehensive Analysis of Common Serum Liver Enzymes as Prospective Predictors of Hepatocellular Carcinoma in HBV Patients
Source: PLoS One. 2012 Oct 24;7(10):e47687. doi: 10.1371/journal.pone.0047687 (PMC3480412; doi:10.1371/journal.pone.0047687)
Supplement: Table S3 — The association of serum liver enzyme levels within 2 years of follow-up and HCC risk in HBV-infected patients. (DOCX) [file pone.0047687.s003.docx]

| **Supplementary Table S3. The association of serum liver enzyme levels within 2 years of follow-up and HCC risk in HBV-infected patients** | | | | | | | | | | |
| --- | --- | --- | --- | --- | --- | --- | --- | --- | --- | --- |
| Enzymes | Serum enzyme level status^1^ | Cases/total | Univariate | |  | Multivariate-adjusted^2^ | |  | Multivariate-adjusted^3^ | |
|  |  |  | HR (95% CI) | *P* value |  | HR (95% CI) | *P* value |  | HR (95% CI) | *P* value |
| ALT | By average in first 2 years of follow-up | | | | | | | | | |
|  | Normal | 15/226 | 1 |  |  | 1 |  |  | 1 |  |
|  | Elevated | 37/362 | 1.32(0.72-2.42) | 0.363 |  | 1.07(0.55-2.06) | 0.848 |  | 1.41(0.74-2.66) | 0.296 |
|  | By maximum in first 2 years of follow-up | | | | | | | | | |
|  | Normal | 12/167 | 1 |  |  | 1 |  |  | 1 |  |
|  | Elevated | 40/421 | 1.31(0.69-2.52) | 0.409 |  | 0.90(0.44-1.85) | 0.771 |  | 1.40(0.71-2.76) | 0.336 |
| AST | By average in first 2 years of follow-up | | | | | | | | | |
|  | Normal | 18/290 | 1 |  |  | 1 |  |  | 1 |  |
|  | Elevated | 34/298 | 1.60(0.90-2.84) | 0.112 |  | 0.96(0.51-1.80) | 0.899 |  | 1.45(0.80-2.66) | 0.223 |
|  | By maximum in first 2 years of follow-up | | | | | | | | | |
|  | Normal | 14/228 | 1 |  |  | 1 |  |  | 1 |  |
|  | Elevated | 38/360 | 1.72(0.93-3.18) | 0.085 |  | 1.01(0.51-2.01) | 0.969 |  | 1.64(0.86-3.12) | 0.132 |
| ALP | By average in first 2 years of follow-up | | | | | | | | | |
|  | Normal | 38/508 | 1 |  |  | 1 |  |  | 1 |  |
|  | Elevated | 14/80 | **3.78(2.01-7.08)** | **<0.001** |  | 1.79(0.90-3.55) | 0.098 |  | **2.43(1.26-4.71)** | **0.008** |
|  | By maximum in first 2 years of follow-up | | | | | | | | | |
|  | Normal | 34/440 | 1 |  |  | 1 |  |  | 1 |  |
|  | Elevated | 18/148 | **2.34(1.30-4.22)** | **0.005** |  | 1.33(0.72-2.45) | 0.362 |  | **1.86(1.02-3.39)** | **0.042** |
| GGT | By average in first 2 years of follow-up | | | | | | | | | |
|  | Normal | 17/388 | 1 |  |  | 1 |  |  | 1 |  |
|  | Elevated | 35/200 | **3.71(2.07-6.64)** | **<0.001** |  | **2.25(1.20-4.21)** | **0.011** |  | **2.55(1.37-4.74)** | **0.003** |
|  | By maximum in first 2 years of follow-up | | | | | | | | | |
|  | Normal | 13/328 | 1 |  |  | 1 |  |  | 1 |  |
|  | Elevated | 39/260 | **3.41(1.82-6.41)** | **<0.001** |  | 1.86(0.93-3.72) | 0.081 |  | **2.26(1.15-4.45)** | **0.018** |
| Notes: ^1^The cutoff values for ALT are: Normal, ALT ≤ 40.0 U/L for male or ≤ 31.0 U/L for female; Elevated, ALT > 40.0 U/L for male or > 31.0 U/L for female; the cutoff values for AST are: Normal, AST ≤ 37.0 U/L for male or ≤ 31.0 U/L for female; Elevated, AST > 37.0 U/L for male or > 31.0 U/L for female; the cutoff values for ALP are: Normal, ALP ≤ 117.0 U/L for all patients; Elevated, ALP > 117.0 U/L for adults and 117-390 for children (3-15 years); the cutoff values for GGT are: Normal, GGT ≤ 51.0 U/L for male or GGT ≤ 33.0 U/L for female; Elevated, GGT > 51.0 U/L for male or > 33.0 U/L for female. ^2^HR adjusted for gender, age, smoking status, alcohol consumption, cirrhosis, and family cancer. ^3^HR adjusted for gender, age, smoking status, alcohol consumption, and family cancer. | | | | | | | | | | |
|  |  |  |  |  |  |  |  |  |  |  |
|  |  |  |  |  |  |  |  |  |  |  |
|  |  |  |  |  |  |  |  |  |  |  |
