# Supplementary material for: Comprehensive Analysis of Common Serum Liver Enzymes as Prospective Predictors of Hepatocellular Carcinoma in HBV Patients
Source: PLoS One. 2012 Oct 24;7(10):e47687. doi: 10.1371/journal.pone.0047687 (PMC3480412; doi:10.1371/journal.pone.0047687)
Supplement: Table S4 — The association of serum GGT levels and HCC risk in HBV-infected patients with dose-dependent manner. (DOCX) [file pone.0047687.s004.docx]

| **Supplementary Table S4. The association of serum GGT levels and HCC risk in HBV-infected patients with dose-dependent manner** | | | | | | | |
| --- | --- | --- | --- | --- | --- | --- | --- |
| Serum GGT status^1^ | Number of patients (n=588) | Number of HCC cases (n=52) | Unadjusted | |  | Multivariate-adjusted^2^ | |
|  |  |  | HR (95% CI) | *P* value |  | HR (95% CI) | *P* value |
| By baseline |  |  |  |  |  |  |  |
| Normal | 374 | 17 | 1.00 |  |  | 1.00 |  |
| Elevated | 121 | 19 | **3.72(1.93-7.19)** | **<0.001** |  | **3.01(1.49-6.11)** | **0.002** |
| Highly elevated | 93 | 16 | **4.16(2.09-8.29)** | **<0.001** |  | **2.26(1.11-4.62)** | **0.024** |
| *P* trend |  |  |  | **< 0.001** |  |  | **0.012** |
| By average in first 1 year follow-up | |  |  |  |  |  |  |
| Normal | 374 | 16 | 1.00 |  |  | 1.00 |  |
| Elevated | 129 | 19 | **3.38(1.73-6.60)** | **<0.001** |  | 1.44(0.66-3.11) | 0.357 |
| Highly elevated | 85 | 17 | **4.10(2.06-8.16)** | **<0.001** |  | **2.81(1.38-5.72)** | **0.004** |
| *P* trend |  |  |  | **< 0.001** |  |  | **0.005** |
| By maximum in first 1 year follow-up | |  |  |  |  |  |  |
| Normal | 337 | 13 | 1.00 |  |  | 1.00 |  |
| Elevated | 124 | 18 | **3.61(1.76-7.38)** | **<0.001** |  | 1.65(0.74-3.71) | 0.222 |
| Highly elevated | 127 | 21 | **3.65(1.82-7.31)** | **<0.001** |  | **2.17(1.04-4.52)** | **0.039** |
| *P* trend |  |  |  | **< 0.001** |  |  | **0.039** |
| By average in first 2 years follow-up | |  |  |  |  |  |  |
| Normal | 388 | 17 | 1.00 |  |  | 1.00 |  |
| Elevated | 116 | 16 | **3.17(1.59-6.32)** | **0.001** |  | 1.60(0.74-3.45) | 0.229 |
| Highly elevated | 84 | 19 | **4.31(2.23-8.34)** | **0.000** |  | **2.94(1.48-5.82)** | **0.002** |
| *P* trend |  |  |  | **<0.001** |  |  | **0.002** |
| By maximum in first 2 years follow-up | |  |  |  |  |  |  |
| Normal | 328 | 13 | 1.00 |  |  | 1.00 |  |
| Elevated | 124 | 16 | **3.14(1.51-6.55)** | **0.002** |  | 1.52(0.67-3.45) | 0.311 |
| Highly elevated | 136 | 23 | **3.63(1.83-7.19)** | **<0.001** |  | **2.10(1.01-4.35)** | **0.047** |
| *P* trend |  |  |  | **<0.001** |  |  | **0.043** |
| Notes:^1^The cutoff values are: Normal, GGT ≤ 51.0 U/L for men or ≤ 33.0 U/L for women; Elevated, 102.0 ≥ GGT > 51.0 U/L for men or 66.0 ≥ GGT > 33.0 U/L for women; Highly elevated, GGT> 102.0 U/L for men or > 66.0 U/L for women. ^2^HR adjusted for gender, age, smoking status, drinking status, cirrhosis, and family cancer. | | | | | | | |
