# Supplementary material for: Comprehensive Analysis of Common Serum Liver Enzymes as Prospective Predictors of Hepatocellular Carcinoma in HBV Patients
Source: PLoS One. 2012 Oct 24;7(10):e47687. doi: 10.1371/journal.pone.0047687 (PMC3480412; doi:10.1371/journal.pone.0047687)
Supplement: Table S5 — The association of baseline GGT levels and the risk of HCC stratified by demographic variables. (DOCX) [file pone.0047687.s005.docx]

| **Supplementary Table S5. The association of baseline GGT levels and the risk of HCC stratified by demographic variables** | | | | | | | | |
| --- | --- | --- | --- | --- | --- | --- | --- | --- |
| Variables | Serum enzyme levels^1^ | Number of patients | Number of cases | Unadjusted | |  | Multivariate-adjusted^2^ | |
|  |  |  |  | HR (95%CI) | *P* value |  | HR (95%CI) | *P* value |
| Gender |  |  |  |  |  |  |  |  |
| Female | Normal | 128 | 1 | 1.00 |  |  | 1.00 |  |
|  | Elevated | 60 | 7 | **13.8(1.68- 113)** | **0.015** |  | 4.41(0.51-38.4) | 0.179 |
| Male | Normal | 246 | 16 | 1.00 |  |  | 1.00 |  |
|  | Elevated | 154 | 28 | **3.03(1.64-5.62)** | **< 0.001** |  | **2.48(1.29-4.75)** | **0.006** |
| Age |  |  |  |  |  |  |  |  |
| Younger | Normal | 207 | 6 | 1.00 |  |  | 1.00 |  |
|  | Elevated | 87 | 5 | 2.42(0.73-8.01) | 0.148 |  | 3.77(1.00-14.3) | 0.051 |
| Older | Normal | 167 | 11 | 1.00 |  |  | 1.00 |  |
|  | Elevated | 127 | 30 | **3.73(1.81-7.68)** | **< 0.001** |  | **3.11(1.46-6.63)** | **0.003** |
| Smoking status | |  |  |  |  |  |  |  |
| Never | Normal | 276 | 9 | 1.00 |  |  | 1.00 |  |
|  | Elevated | 130 | 19 | **4.90(2.20-10.9)** | **< 0.001** |  | **2.81(1.17-6.75)** | **0.021** |
| Ever | Normal | 98 | 8 | 1.00 |  |  | 1.00 |  |
|  | Elevated | 84 | 16 | **2.67(1.14-6.26)** | **0.024** |  | **3.41(1.32-8.77)** | **0.011** |
| Alcohol consumption | |  |  |  |  |  |  |  |
| Never | Normal | 251 | 9 | 1.00 |  |  | 1.00 |  |
|  | Elevated | 121 | 19 | **4.13(1.86-9.17)** | **< 0.001** |  | **2.43(1.06-5.58)** | **0.036** |
| Ever | Normal | 123 | 8 | 1.00 |  |  | 1.00 |  |
|  | Elevated | 93 | 16 | **3.42(1.40-8.32)** | **0.007** |  | **4.05(1.51-10.9)** | **0.006** |
| Cirrhosis |  |  |  |  |  |  |  |  |
| No | Normal | 271 | 3 | 1.00 |  |  | 1.00 |  |
|  | Elevated | 111 | 5 | **5.25(1.02-27.1)** | **0.048** |  | NA |  |
| Yes | Normal | 103 | 14 | 1.00 |  |  | 1.00 |  |
|  | Elevated | 103 | 30 | **3.23(1.69-6.19)** | **< 0.001** |  | **2.77(1.41-5.46)** | **0.003** |
| Family cancer |  |  |  |  |  |  |  |  |
| No | Normal | 246 | 10 | 1.00 |  |  | 1.00 |  |
|  | Elevated | 146 | 22 | **4.18(1.97-8.87)** | **< 0.001** |  | **2.69(1.22-5.96)** | **0.014** |
| Yes | Normal | 128 | 7 | 1.00 |  |  | 1.00 |  |
|  | Elevated | 68 | 13 | **3.71(1.40-9.80)** | **0.008** |  | 2.43(0.81-7.27) | 0.113 |
| Notes: ^1^The cutoff values for GGT are: Normal, GGT ≤ 51.0 U/L for male or GGT ≤ 33.0 U/L for female; Elevated, GGT > 51.0 U/L for male or > 33.0 U/L for female; ^2^Adjusted for gender, age, smoking status, drinking status, cirrhosis, and family cancer, where appropriately | | | | | | | | |
