# Supplementary material for: Comprehensive Analysis of Common Serum Liver Enzymes as Prospective Predictors of Hepatocellular Carcinoma in HBV Patients
Source: PLoS One. 2012 Oct 24;7(10):e47687. doi: 10.1371/journal.pone.0047687 (PMC3480412; doi:10.1371/journal.pone.0047687)
Supplement: Table S6 — Cumulative incidence of HCC by serum liver enzymes at baseline levels and different years of follow-up. (DOCX) [file pone.0047687.s006.docx]

| **Supplementary Table S6. Cumulative incidence of HCC by serum liver enzymes at baseline levels and different years of follow-up** | | | | | | |
| --- | --- | --- | --- | --- | --- | --- |
| Years of follow-up (y) | Cumulative incidence (95% CI) | | | | | |
|  | ALT^1^ | |  | AST^2^ | |  |
|  | Normal | Elevated |  | Normal |  |  |
| 3 | 0.9 (0.2-3.6) | 0.9 (0.3-2.8) |  | 0.3 (0-2.4) |  |  |
| 6 | 3.5 (1.6-7.9) | 4.8 (2.7-8.3) |  | 2.4 (1.0-5.7) |  |  |
| 9 | 8.4 (4.4-15.7) | 6.7 (4.0-11.1) |  | 5.9 (3.0-11.4) |  |  |
| 12 | 11.6 (6.4-20.7) | 15.3 (9.9-23.1) |  | 9.4 (5.2-16.7) |  |  |
| 15 | 16.8 (9.4-29.0) | 26.5 (17.7-38.6) |  | 15.5 (8.9-26.2) |  |  |
| >18 | 44.0 (22.4-73.4) | 77.3 (42.0-98.2) |  | 69.9 (30.3-98.2) |  |  |
| Note: ^1^The cutoff values for ALT are: Normal, ALT ≤ 40.0 U/L for male or ≤ 31.0 U/L for female; Elevated, ALT > 40.0 U/L for male or > 31.0 U/L for female; ^2^the cutoff values for AST are: Normal, AST ≤ 37.0 U/L for male or ≤ 31.0 U/L for female; Elevated, AST > 37.0 U/L for male or > 31.0 U/L for female; ^3^the cutoff values for ALP are: Normal, ALP ≤ 117.0 U/L for all patients; Elevated, ALP > 117.0 U/L for all patients; ^4^the cutoff values for GGT are: Normal, GGT ≤ 51.0 U/L for male or GGT ≤ 33.0 U/L for female; Elevated, GGT > 51.0 U/L for male or > 33.0 U/L for female. | | | | | | |
